# Using Electronic Health Record Data for Healthy Weight Surveillance in Children, San Diego, California, 2014

**DOI:** 10.5888/pcd13.150422

**Published:** 2016-03-10

**Authors:** Ashley M. Kranz, Deirdre K. Browner, Lindsey McDermid, Thomas R. Coleman, Wilma J. Wooten

**Affiliations:** Author Affiliations: Deirdre K. Browner, Lindsey McDermid, Thomas R. Coleman, Wilma J. Wooten, County of San Diego Health and Human Services Agency, Public Health Services, San Diego, California.

**Figure:**
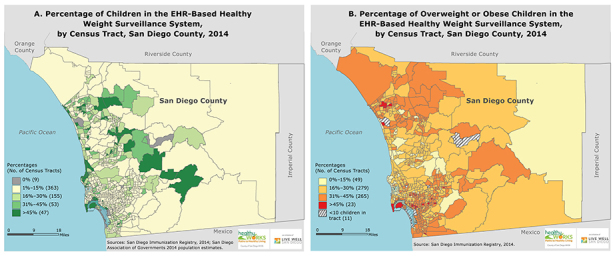
The San Diego EHR-Based Healthy Weight Surveillance System includes, on average, 18.5% (standard deviation, 19.4%) of children per census tract. These preliminary, nonrepresentative data illustrate geographic variation in the prevalence of overweight and obese children. The maps demonstrate both the strengths and the challenges of using EHR-based data for surveillance. Abbreviation: EHR, electronic health record.

## Background

The expanded use of electronic health records (EHRs) provides new opportunities to monitor population health at the local level. Meaningful use objectives in the Medicare and Medicaid EHR incentive programs promote public health departments’ use of EHR data for establishing registries, conducting syndromic surveillance, and monitoring reportable conditions. Additionally, the federal government illustrated the use of aggregated EHR data, through electronic clinical quality measures (eCQMs), to assess blood pressure control and encouraged further use of eCQMs for population health surveillance (1). Use of EHR data for chronic disease surveillance by public health departments has been encouraged because of the potential of these data to provide timely, geographically specific, and clinically detailed health information that can be used to better understand the distribution of disease, measure health disparities, and promote public health (1–5).

In 2010, San Diego County used funds from a Communities Putting Prevention to Work award to develop a healthy weight surveillance system to collect measures of height and weight from EHRs. Because existing surveys provided self-reported height and weight (eg, via the California Health Interview Survey) or estimates not generalizable to neighborhoods (eg, via the National Health and Nutrition Examination Survey), San Diego sought EHR data to track progress of local efforts to reduce obesity and inform community outreach activities. Using the county’s immunization registry as a vehicle for electronic data transfer, measures of height and weight were collected for more than 700,000 people from 6 community health clinics, 3 large medical systems, and 3 private health clinics (6). This article illustrates the benefits and challenges of using EHR data for healthy weight surveillance and public health planning.

## Methods

We examined measures of height and weight obtained from children at medical visits, including nonimmunization visits, during the 2014 calendar year. We identified body mass index (BMI)-for-age percentiles for children aged 2 to 18 years, excluding biologically implausible values, by using an anthropometric SAS program (SAS Institute, Inc) from the Centers for Disease Control and Prevention (CDC). BMI values were categorized as overweight or obese by using age-specific percentile growth charts for children. If children had multiple measures of height and weight, the most recent were used. We used ArcMap 10.2 (Esri) to geocode children’s home street addresses (match rate = 95%) and then aggregated these points to census tracts. We eliminated from analysis census tracts with fewer than 10 children (n = 11) in the healthy weight surveillance system to maintain privacy. 

To assess coverage of the healthy weight surveillance system, we used 2014 population estimates obtained from the San Diego Association of Governments and divided the number of children with a valid BMI measurement within a census tract by the 2014 total population of children aged 2 to 18 years, in each of the county’s 627 census tracts. To assess prevalence of overweight and obesity, we divided the number of children identified as overweight or obese by the total number of children included in the healthy weight surveillance system. Coverage of the healthy weight surveillance system and prevalence of child overweight and obesity in the healthy weight surveillance system are illustrated by census tract in the maps.

## Main Findings

In 2014, the County of San Diego healthy weight surveillance system included 106,717 children aged 2 to 18 years, covering 14.5% of the county’s child population. On average, our sample (male = 51.4%, non-Hispanic white = 29.1%, Hispanic = 44.9%) differed somewhat from the full county child population (male = 51.5%, non-Hispanic white = 36.7%, Hispanic = 43.3%). System coverage varied. On average, the healthy weight surveillance system included 18.5% (standard deviation = 19.4%) of children per census tract. Only 16% of census tracts (n = 100) had more than 30% of their child population covered by the healthy weight surveillance system, which highlights the need to expand data collection efforts and continue to assess the representativeness of existing data.

Among children included in the healthy weight surveillance system, 31.7% were overweight or obese, which is slightly less than the rate observed from San Diego County children participating in the school-based 2010 California Physical Fitness Test (34.5%) (7). Most census tracts (n = 328) had 30% or fewer children who were overweight or obese. The percentage of overweight or obese children per census tract ranged from 0% to 55%, suggesting geographic variation in rates of child overweight and obesity throughout San Diego County.

## Action

Using data from EHRs has been proposed as a way to monitor population health at the local level. Whereas surveys may rely on self-reported information or lack detail about small geographic areas, EHR data in San Diego County provided measures of child overweight and obesity that were timely, geographically detailed, and clinically valid. However, because these data included only a fraction of the county’s pediatric population, more work is needed to improve and assess the representativeness of these data for them to be used to make conclusions about population health and track health disparities.

Challenges remain to using EHR data for surveillance activities. First, these data represent a convenience sample of people with medical encounters, which misses people without medical encounters, a population of great interest to local health departments. Furthermore, as in San Diego County’s experience, not all medical clinics may share data, introducing additional bias because these data exclude both people who receive medical care from nonparticipating clinics and people without medical encounters. Obtaining engagement from medical groups can be challenging and requires continued effort to ensure that groups share data over time as technology, priorities, and leadership change. These limitations highlight the need to expand data collection efforts, assess the representativeness of the EHR data collected for surveillance activities, and refine methods of adjustment for nonrepresentative samples.

Despite these limitations, EHRs continue to be an innovative data source for local health departments to explore how obesity and other health outcomes vary across neighborhoods, identify health disparities, and target limited resources accordingly (4–6,8). As the adoption and technical capacity of EHRs expand, local health departments should consider using geographic information system methods and EHR data, at the individual level or aggregated to eCQMS, to conduct population health surveillance and inform the planning of health promotion activities.
